# Warm Reception? Halogenated BPA Flame Retardants and PPARγ Activation

**DOI:** 10.1289/ehp.119-a398b

**Published:** 2011-09-01

**Authors:** Julia R. Barrett

**Affiliations:** Julia R. Barrett, MS, ELS, a Madison, WI–based science writer and editor, has written for *EHP* since 1996. She is a member of the National Association of Science Writers and the Board of Editors in the Life Sciences.

Adverse effects highlighted in experimental studies of the industrial chemical bisphenol A (BPA) have centered on reproductive targets, but recent research suggests metabolism also may be affected. BPA is widely present in the environment, as are its halogenated derivatives. A new study investigates the structure and function of these halogenated compounds and shows they could potentially disrupt energy balance (i.e., lipid and carbohydrate homeostasis) in humans and wildlife [*EHP* 119(9):1227–1232; Riu et al.].

Halogenated BPA derivatives are used as flame retardants, with tetrabromobisphenol A (TBBPA) and tetrachlorobisphenol A (TCBPA) as parent compounds. Previous studies have shown that BPA, TBBPA, and TCBPA have estrogenic activity and that BPA can alter adipogenesis (fat cell formation) in rats. Peroxisome proliferator-activated receptor γ (PPARγ) plays a key role in adipogenesis, and interference with this receptor can contribute to type 2 diabetes and obesity.

Cell lines were used to test the ability of BPA, TBBPA, TCBPA, and other halogenated BPA derivatives to activate human estrogen receptors (ERα and ERβ) and human PPARs (PPARα, PPARδ, and PPARγ). The compounds also were applied to undifferentiated fat cells to test their effects on adipogenesis and the genes involved in this process. Competitive binding assays were conducted to further characterize PPARγ activation in both reporter cell lines and undifferentiated fat cells, and crystals of PPARγ bound to TBBPA and TCBPA were purified to study how the chemicals bound and activated the receptor. Another set of experiments used *Xenopus* and zebrafish PPARγ to test the response to BPA compounds in other species.

TCBPA and lower brominated BPAs such as monoBBPA and diBBPA partially activated both ER subtypes, whereas TBBPA had little effect on either. Both TBBPA and TCBPA were able to partially activate PPARγ and promote fat cell differentiation as well as disrupt PPARγ activity in all species studied. Structural examination of the area where compounds are bound to the PPARγ receptor yielded evidence that TBBPA, TCBPA, and other halogenated BPA derivatives partially activate this receptor.

What does all this mean? Essentially, the findings support the hypothesis that environmental contaminants may contribute to the disruption of energy balance in humans and wildlife. It also suggests that halogenated derivatives of BPA used as flame retardants, which have not been extensively studied to date, may contribute to obesity. The main active form of PPARγ is the retinoid X receptor (RXR)/PPARγ heterodimer. According to the authors, environmental exposure to BPA compounds alongside other endocrine disruptors with similar RXR and/or PPARγ activity sets the stage for additive and synergistic effects, potentially leading to increased risk of metabolic disease.

